# Sliding mode-based direct power control of unified power quality conditioner

**DOI:** 10.1016/j.heliyon.2024.e39597

**Published:** 2024-10-21

**Authors:** Tapankumar Trivedi, Rajendrasinh Jadeja, Praghnesh Bhatt, Chao Long, P. Sanjeevikumar, Amit Ved

**Affiliations:** a Department of Electrical Engineering, Marwadi University, Rajkot-Morbi Highway, Rajkot, 360003, Gujarat, India; b School of Technology, Pandit Deendayal Energy University, Knowledge Corridor, Raisan Village, Raisan, Gandhinagar, 382007, Gujarat, India; c Department of Electrical Engineering and Electronics, University of Liverpool, Liverpool, L69 3GJ, UK; d Department of Electrical Engineering, IT and Cybernetics, University of South-Eastern, Kjølnes Ring 56, Porsgrunn, 3918, Norway

**Keywords:** DC-AC converter, Direct power control, Harmonics, Power quality, Unified power quality conditioner

## Abstract

The Unified Power Quality Conditioner (UPQC) is a promising solution for mitigating multiple Power Quality(PQ) issues in distribution systems, including harmonics, poor power factor, voltage sag/swell and voltage imbalance. The conventional Sliding Mode Controller (SMC) in UPQCs suffers from wide switching frequency variations, chattering problems, and inherent active and reactive power coupling. This study proposes a nonlinear control method, Sliding Mode-based Direct Power Control (SMC-DPC), for the simultaneous regulation of the shunt and series compensators in a UPQC. By optimizing voltage vector selection based on real-time power errors, the proposed method effectively mitigates chattering, and reduces switching frequency variations, and ensures precise tracking of instantaneous active and reactive powers even in the presence of coupling effects. The proposed approach simplifies the system design and improves steady state and dynamic power tracking. The simulation results in MATLAB Simulink® on a 20 kVA system demonstrate that the proposed method achieves a source current THD of 1.52%, compared to 2.31% for conventional SMC and 5.11% for linear controller. Furthermore, the method is robust to grid parameter variations and demonstrates satisfactory performance in both strong and weak grids. In weak grids, the proposed method reduces the line losses by 13.71% and 14.54% compared to SMC and linear controller respectively. The reported results comply with international standards such as IEEE-519 and IEC 61000-3-12, confirming effectiveness of SMC-DPC for enhancing PQ in distribution system.

## Nomenclature

α: Component along *α*-axis in Stationary Reference Frame
β: Component along *β*-axis in Stationary Reference Frame
$\overset{˙}{x}$: First Order Derivative of Quantity
$\eta_{conv}$: Efficiency of Converter
$V_{i}$: $i^{th}$ Voltage Vector of Converter of Compensator
$\omega_{e}$: Angular Frequency of Grid Supply
$\omega_{0}$: Tuned Frequency of Positive Sequence Estimator
$\Theta_{i}$: $i^{th}$ sector of Voltage/Current Vector in Hexagonal Plane
$\theta_{e}$: Grid Voltage Angle
$\overset{ˆ}{}$: Estimated Quantity in Stationary Reference Frame
Cse: Coupling Capacitor of Series Compensator
hp/q: Width of the Boundary Layer for Instantaneous Power Error
$i_{f\alpha }$: Component of Shunt Compensator Current Vector on *α*-axis
$i_{f\beta }$: Component of Shunt Compensator Current Vector on *β*-axis
$I_{h}$: RMS value of harmonic component of load
$I_{p,sag}$: RMS value of current requirement during sag
$I_{q}$: RMS value of reactive power component of current
$i_{s\alpha }$: Component of Source Current Vector on *α*-axis
$i_{s\beta }$: Component of Source Current Vector on *β*-axis
$i_{se\alpha }$: Component of Series Compensator Current Vector on *α*-axis
$i_{se\beta }$: Component of Series Compensator Current Vector on *β*-axis
Is: RMS Value of Source Current
kp: Sliding Coefficient for Instantaneous Active Power of Shunt Compensator
kq: Sliding Coefficient for Instantaneous Reactive Power of Shunt Compensator
ku: Sliding Coefficient for Instantaneous Active Power Series Compensator
kv: Sliding Coefficient for Instantaneous Reactive Power Series Compensator
$K_{\sigma }$: Sharpness Constant of Positive Sequence Estimator
kid: Integral Constant of DC link Voltage Controller
kpd: Proportional Constant of DC link Voltage Controller
Lf: Coupling inductor of Shunt Compensator
$L_{s}$: Inductance of Grid
Lse: Coupling Inductor of Series Compensator
ma: Modulation Index of Converter of Shunt Compensator
n: No. of fundamental cycles of supply to restore DC link
pL: Instantaneous Active Power of Load
pdc: Instantaneous Active Power Demanded by DC link
Pgl: Real Power Losses in Grid
ploss: Losses of Compensators and Coupling Filters
pse: Instantaneous Active Power of Series Compensator
psh: Instantaneous Active Power of Shunt Compensator
Psw: switching losses of both converters
qL: Instantaneous Reactive Power of Load
qse: Instantaneous Reactive Power of Series Compensator
qsh: Instantaneous Reactive Power of Shunt Compensator
rf: Resistance of the Coupling Inductor of Shunt Compensator
$r_{s}$: Resistance of Grid
Si: Switching States of Converter of Compensator
Sp: Sliding Surface of Instantaneous Active Power of Shunt Compensator
Sq: Sliding Surface of Instantaneous Reactive Power of Shunt Compensator
Su: Sliding Surface of Instantaneous Active Power Series Compensator
Sv: Sliding Surface of Instantaneous Reactive Power Series Compensator
Sse: Apparent Power of Series Compensator
Ssh: Apparent Power of Shunt Compensator
V(t): Lyapunov Function
Vdc: DC link Voltage of UPQC
$v_{inj\alpha }$: Component of Injected Voltage Vector of Series Compensator on *α*-axis
$v_{inj\beta }$: Component of Injected Voltage Vector of Series Compensator on *β*-axis
$v_{L\alpha }$: Component of Load Voltage Vector on *α*-axis
$v_{L\beta }$: Component of Load Voltage Vector on *α*-axis
VLL: RMS Value of Line to Line Voltage at Point of Common Coupling
Vph: RMS Value of Phase Voltage at Point of Common Coupling
$v_{s\alpha }$: Component of Source Voltage Vector on *α*-axis
$v_{s\beta }$: Component of Source Voltage Vector on *β*-axis
$v_{se\alpha }$: Component of Series Compensator Voltage Vector on *α*-axis
$v_{se\beta }$: Component of Series Compensator Voltage Vector on *β*-axis
$v_{sh\alpha }$: Component Shunt Compensator Voltage Vector on *α*-axis
$v_{sh\beta }$: Component Shunt Compensator Voltage Vector on *β*-axis
$x^{⁎}$: Reference Value of Quantity

**Acronyms**
**ANN**: artificial neural network
**ANFIS**: artificial neuro-fuzzy interface system
**ESOGI**: enhanced second order generalized integrator
**FCS**: finite control set
**FFT**: fast Fourier transform
**HCC**: hyteresis current controller
**HVC**: hyteresis voltage controller
**IARP**: instantaneous active and reactive power
**ICCS**: independent current compensation strategy
**LMBP**: Levenberg–Marquardt backpropagation
**LUnCS**: load unbalance compensation strategy
**LPF**: low pass filter
**LVRT**: low voltage ride through
**mFLL**: modified frequency locked loop
**MPC**: model predictive controller
**PI**: proportional integral
**PI-MR**: proportional integral multiresonant
**PLL**: phase locked loop
**PPF - EHSA**: predator prey firefly and enhanced harmony search optimization
**PR**: proportional resonant
**PSMC**: passive sliding mode control
**SF-MR**: state feedback multiresonant
**SMC**: sliding mode control
**SOGI**: second order generalized integrator
**SRF**: synchronous reference frame
**SWFA**: sliding window Fourier analysis
**THD**: total harmonic distortion
**UPQC**: unified power quality conditioner
**UVT**: unit vector template
**VSI**: voltage source inverter
**WECS**: wind energy conversion system

## 1 Introduction

In recent years, the power grid is transforming quickly from the centralized grid to distributed Renewable Energy Systems (RES) and energy storage system based grids. The contribution of renewable energy has increased significantly in developing countries to meet growing energy demands. The grid-connected inverters which are paired with DC-DC converters [1], [2] for RES, are designed to provide satisfactory performance under rated power condition [3], but their operation is often overlooked at partial load. Consequently, a considerable amount of harmonics is injected into the grid, increasing power system losses, particularly when PV and/or WECS are connected through multiple voltage source converters.

Furthermore, adjustable speed drives used to improve industrial energy efficiency are found to have detrimental effects on the distribution system due to the nonlinear behavior of the AC/DC converter. Additionally, the sudden switching of de-energized capacitors in switching power supplies and battery chargers used in hybrid energy systems and Electric Vehicles (EVs) causes large inrush currents, leading to sudden voltage dips. In the event of recurrent voltage sags, sensitive equipment in the industry must be protected to prevent failure. Adding to these challenges is the increasing adoption of EVs, which presents a significant challenge to power grid stability. While EVs contribute to a greener transportation sector, their charging behavior, particularly uncontrolled charging during peak hours, can amplify existing PQ issues [4]. This necessitates sophisticated grid management solutions to mitigate the negative impacts and harness the potential benefits of widespread EV adoption. These issues encountered in modern distribution systems can be addressed by PQ improvement devices. A shunt compensator is used to mitigation PQ issues such as current harmonics, poor power factor, and load unbalance [5]. On the other hand, series compensator is used to mitigate voltage unbalance, sag/swell, voltage harmonics or interruptions [5]. However, multiple PQ issues in the modern distribution system can only be addressed by a PQ mitigation device capable of solving two or more of the aforementioned problems simultaneously such as UPQC [6].

**Figure 1:**
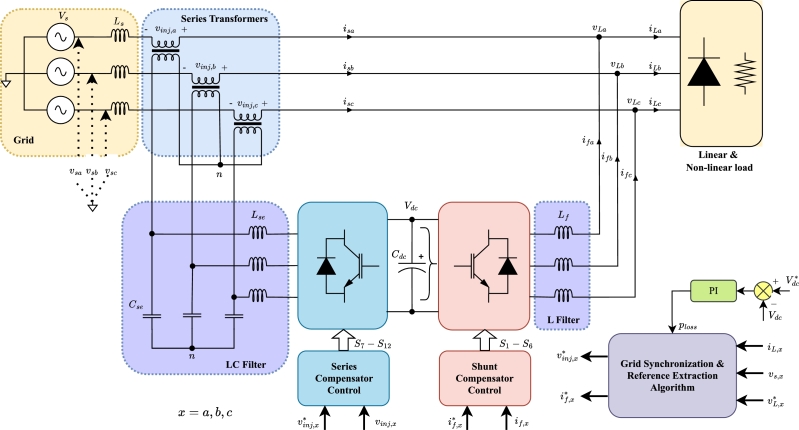
Power Circuit Diagram of UPQC.

UPQC is a power quality improvement solution that incorporates the salient features of the shunt compensator and the series compensator. The power circuit diagram of the UPQC is shown in Fig. 1. The shunt compensator injects the current to compensate harmonics and reactive power generated by a combination of conventional and non-linear loads whereas the series compensator targets the voltage sag, unbalanced, and/or voltage harmonics arising from the source side [7]. The performance of the UPQC depends on the DC link voltage controller, grid synchronization, reference generation algorithm, and converter control. The primary function of the DC link voltage controller is to regulate the DC link voltage to ensure compensation capability of the system. The grid synchronization and reference extraction algorithm accurately determine the reference current of the shunt compensator and the reference voltage of the series compensator. The role of converter controllers is to realize the current and voltage in the shunt compensator and series compensator, respectively. A detailed literature review on each of this is presented in the following section.

## 2 Literature review

In this section, different approaches to improve the performance of UPQC are discussed. Various topologies, DC link voltage control, grid synchronization methods, reference generation algorithm, converter control and an emerging paradigm of RES interface are reported with their merits and limitations.

The type of distribution system and nature of PQ issues determine the topology of UPQC. Various topologies have been proposed in literature, ranging from single-phase H-bridge topology [8], three leg back to back VSI configuration [9] for floating neutral, to three phase four-leg VSI [10] for system with neutral wire. In above reported topologies, both the compensator of UPQC share a common DC link. The magnitude of DC link voltage ascertains compensation capability of UPQC. This necessitates the control of DC link voltage under different conditions.

The DC link voltage controller ensures the compensation capability of the UPQC, determining the overall system response while ensuring robustness and stability. The PI controller, referenced in [11], has been widely used in various implementations. However, PI controllers are sensitive to parameter variations and may exhibit slow dynamic response. Authors in [12] have introduced feed-forward loops to enhance the dynamic response. A variable nonlinear gain fuzzy controller, as proposed in [13], demonstrates reduced ripple in DC link voltage and superior dynamic performance. However, deciding on an optimal set of fuzzy rules and membership functions remains a challenge in the controller. Hence, ANFIS-based controller [14] is proposed that adapts to system parameter variations. A hybrid algorithm [15] as combination of harmonics search algorithm and firefly algorithm is implemented for the selection of optimal parameters of ANFIS based controller. The controller is used for the regulation of UPQC DC link which has interface of PV and battery storage system. In contrast to an FLC-based controller, this approach offers a more structured method and higher accuracy.

Accurate and rapid grid synchronization is crucial for the seamless operation of the UPQC, particularly in dynamic grid environment i.e. fluctuations in source voltage, faults in adjacent feeders, and grid parameters. While traditional SRF-PLL [16] and Negative Feedback SRF-PLL [13] are commonly employed, their reliance on LPF can lead to sluggish responses under unbalanced or dynamic grid conditions and frequency deviations. To address these limitations, researchers have explored alternative approaches for positive sequence extraction. For instance, authors in [17] proposed an ESOGI-based approach for solar PV-fed UPQCs for improved dynamic response. Similarly, [18] introduced a reduced-sensor approach using ESOGI with an mFLL, that eliminates DC offset and reduces harmonic components. Beyond ESOGI, other innovative techniques have been investigated, including the GI-BPF-FLL presented in [19]. This method combines a GI-BPF for effective harmonic extraction with an FLL for precise frequency tracking, enabling robust performance even under distorted grid conditions. Additionally, [20] explored an MAF-based FLL for hydro-turbine systems with adaptability to specific application requirements.

UPQC relies largely on the generation of accurate reference voltage and current signals, which dictate the operation of its shunt and series compensator. Two popular methods widely reported in the literature are SRF theory [21] and the Instantaneous p−q theory [14], each with its own strengths and limitations. Authors in [22] introduce a novel method inspired by Fryze's theory. This approach enables the UPQC to regulate energy flow holistically between sources and loads, rather than solely compensating for non-active current or improving voltage profile. Adaptive intelligence has also found its way into reference signal generation. [23] demonstrates the superior performance of an ANN-based technique, attributed to its adaptability in complex UPQC operating environments. To further improve performance in reference signal generation, researchers in [24] employ SWFA. This method excels in capturing time-varying signals, providing superior frequency resolution and, consequently, improved harmonic identification. In a different application context, [4] utilizes a CLPF based approach for reference signal generation, specifically targeting power quality improvement in EV charging stations.

Integrating RES with UPQC has emerged as a key research area [25], driven by the need to overcome the limitations of traditional UPQC lacking active sources at the DC link. This integration not only enhances power quality but also enables UPQC to act as efficient interfaces for RES [26]. Researchers have explored various configurations and control strategies for this purpose. For instance, [27] demonstrated the autonomous operation of a UPQC integrated with PV and battery storage, while [28] proposed a hybrid-connected UPQC for improved overall system efficiency. Further enhancing performance, [29] optimized parameters for a hybrid AC/DC microgrid with a modulated UPQC. Taking a holistic design approach, [30] utilized an EMVP Algorithm to optimize a PV/Wind/Battery and EV-based UPQC system. Advanced control techniques have also been investigated. [31] presented a novel approach using Football Game Optimization to determine optimal characteristics for a five-level shunt compensator (ShC) controlled by an ANN trained with the LMBP algorithm. Similarly, [32] employed a multi-objective neuro-fuzzy approach, optimizing ANFIS controller parameters using a PPF-EHSA to enhance DC link stability. Beyond grid-connected applications, [33] proposed an interline UPQC to improve the LVRT of WECS, demonstrating the adaptability of UPQC for specific grid requirements.

The converter control governs the performance of UPQC as it synthesizes the injected current and voltages. Furthermore, the choice of control method has impact on steady state and dynamic response, stability of the system, and effectiveness in compensation. Traditional methods like the HCC for shunt compensation and the HVC for series compensation [13], [18], [22], [34] are widely used, but they suffer from significant switching frequency variations, necessitating bulky and costly coupling filters. Linear PI controllers [35], [10], [33], [36] address this drawback by operating at a fixed switching frequency, simplifying filter design and reducing component stress. Despite this advantage, PI controllers are susceptible to harmonic distortions, inherent coupling issues, suboptimal performance with fixed parameters, and sensitivity to parameter variations. Advanced techniques like FCS-MPC [21] offer superior dynamic performance, decoupled control, adaptability, and multi-constraint handling. Nevertheless, computational complexity and model dependency of FCS-MPC pose implementation challenges. PI-MR and SF-MR controllers [8] provide targeted harmonic compensation and enhanced stability. While PI-MR controller is sensitive to grid parameter variations, SF-MR controller exhibits increased complexity and computational demands. PR controllers [34] present a compelling alternative due to their simple implementation and effective targeted harmonic mitigation. It is important to note that PR controllers have limited bandwidth and are susceptible to frequency variations. A summary of all methods reported with different converter control techniques is reported in Table 1.

**Table 1:** An overview of converter control methods of UPQC.

		Control								
		Reference Generation		Converter Control		PQ Issues				Integration of Active Sources
Ref.	Year	Shunt & Series	Synchronization	Shunt	Series	Harmonics	Reactive Power	Sag/ Swell	Unbalance	
[37]	2018	*p* − *q* theory		SMC	SMC	✓	✓	✓	✓	
[35]	2020	SRF	PLL	Linear	Linear	✓	✓	✓	✓	
[8]	2020	Dual Compensation Strategy	1-Φ PLL	SF-MR	PI-MR and SF-MR	✓		✓		
[21]	2020	SRF	PLL	FCS - MPC	FCS - MPC	✓	✓	✓	✓	
[36]	2020	ICCS, LUnCS	1-Φ PLL	Linear	Linear	✓	✓	✓	✓	
[22]	2020	Load Equivalent Conductance	-	HCC	HVC	✓	✓	✓	✓	
[13]	2020	improved SRF	Negative Feedback PLL	HCC	HVC	✓	✓	✓	✓	
[33]	2021	SRF	-	Linear	Linear		✓			Wind, SMES
[18]	2023	ESOGI-mFLL	ESOGI- mFLL	HCC	HVC	✓	✓	✓	✓	PV
[34]	2024	SOGI+PR	SOGI	Linear- PR	Linear- PR	✓		✓		
[10]	2024	Shunt- *pq*-theory, Series- UVT	PLL	Linear	Linear	✓	✓			Battery, PV

Proposed Work		*pq*-theory with adaptive estimation		SMC-DPC	SMC-DPC	✓	✓	✓	✓	

SMC is also a popular non-linear controller which has shown superior performance in UPQC [37]. Various SMC strategies have been explored for DC-DC converters [38], active power control of RES [39] and UPQC. In the UPQC [40], fixed frequency SMC is introduced to eliminate the problem of chattering. A Modular Multilevel Converter based UPQC with PSMC [41] control has shown faster response and robustness against parameter variations. However, these methods have not been examined for the variation in grid parameters. Additionally, all the reported methods in the literature for UPQC are based on the indirect power control strategy which controls the current/voltage in order to regulate the amount of power exchange between grid and compensator. Due to the inherent coupling of active and reactive power, it is difficult to control them precisely leading to poor steady state/dynamic response.

Recently, a nonlinear control strategy namely DPC, a dual of direct torque control method [42], that controls active and reactive power without current control loop [43], [44] is reported for grid connected inverter and shunt compensator [45]. A predictive DPC has been reported for the SAPF application [46]. However, dependency on model parameters makes the method less robust against parameter variations. On the other hand, SMC DPC offers inherent advantages in zero steady state error, decoupled control of power and faster dynamic performance by directly selecting inverter switching states based on instantaneous power errors [47]. By integrating SMC principles into the DPC framework, the proposed method aims to achieve precise and independent control of active and reactive power while mitigating the drawbacks of conventional controllers, such as high switching frequency variations and chattering. This is achieved by reducing the interdependence of phases during voltage vector selection, leading to reduced switching losses and improved overall performance. The main contribution of the article is as follows:

• Development of an SMC-based Direct Power Control (DPC) method that enhances steady-state as well as dynamic performance across various power quality (PQ) issues.
• Optimal vector selection based on instantaneous power errors, designed to eliminate chattering in the non-linear controller and reduce variations in switching frequency.
• Proposal of a modified adaptive frequency algorithm in the stationary reference frame for reference extraction, designed for SMC-based DPC.
• Enhanced robustness to grid parameter variations, ensuring consistent performance under both strong and weak grid conditions.

The proposed method has been compared with SMC controllers, and Linear controller widely employed in the UPQC. With minimal modifications, the proposed method can be extended to several newer approaches to integrating renewable energy sources and electric vehicles (EV) batteries with UPQC into the grid [48], [49], [50].

The paper is structured as follows. In section 3, the concept of sliding mode-based DPC for the control of UPQC compensator is proposed. Since the method requires a separate reference algorithm in the stationary reference frame, a reference algorithm for reference extraction and control of the UPQC is developed in Section 4. The determination of parameters for the UPQC is demonstrated in Section 5. The simulation and performance of the proposed method in comparison to state of the art methods is demonstrated in Section 6 whereas Section 7 reports the performance of the method under Weak and Strong PCC conditions.

## 3 Proposed sliding mode direct power control of UPQC

In both power converters of UPQC i.e. shunt compensator and series compensator, the control input applied to the converter switches is inherently non-linear and contains only two states ($S_{i}=1$ or 0), making it a variable structure system. The prime objective of both converters in UPQC is to deliver the power instantaneously such that any disturbance affecting the system is eliminated by injecting suitable active and reactive powers instantaneously. In Sliding Mode-based DPC, the errors between the reference and actual powers to be controlled are viewed as sliding surfaces. The actual power errors slide along the surface and attain the equilibrium point. If discontinuity borders of IARP errors are divided into $\overset{˙}{p}-\overset{˙}{q}$ subspaces, the existence and uniqueness of the solution is ensured. The suitable control law of UPQC is divided into the following:

1. Control of Shunt Compensator
2. Control of Series Compensator

### 3.1 Control of shunt compensator

The control of the shunt compensator is divided into the modeling of the converter in terms of power variation rates, selection of sliding surface, and determination of the Look-Up Table (LUT) of the voltage vector of the converter.

#### 3.1.1 Modelling of shunt compensator

**Figure 2:**
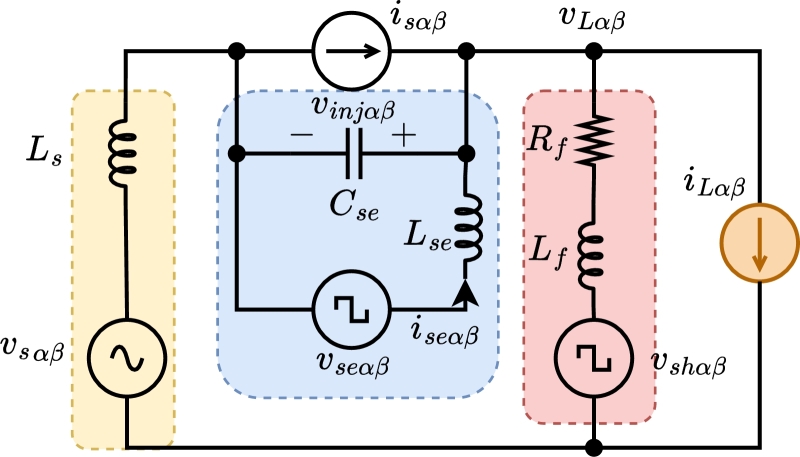
AC equivalent representation of UPQC.

The application of Kirchhoff's Voltage Law (KVL) to the shunt compensator of UPQC, as shown in Fig. 2 yields the following voltage equations,

(1) $$\begin{array}{c} v_{sh\alpha }=r_{f}i_{f\alpha }+L_{f}\overset{˙}{i_{f\alpha }}+v_{L\alpha }\\ v_{sh\beta }=r_{f}i_{f\beta }+L_{f}\overset{˙}{i_{f\beta }}+v_{L\beta } \end{array}$$

**Figure 3:**
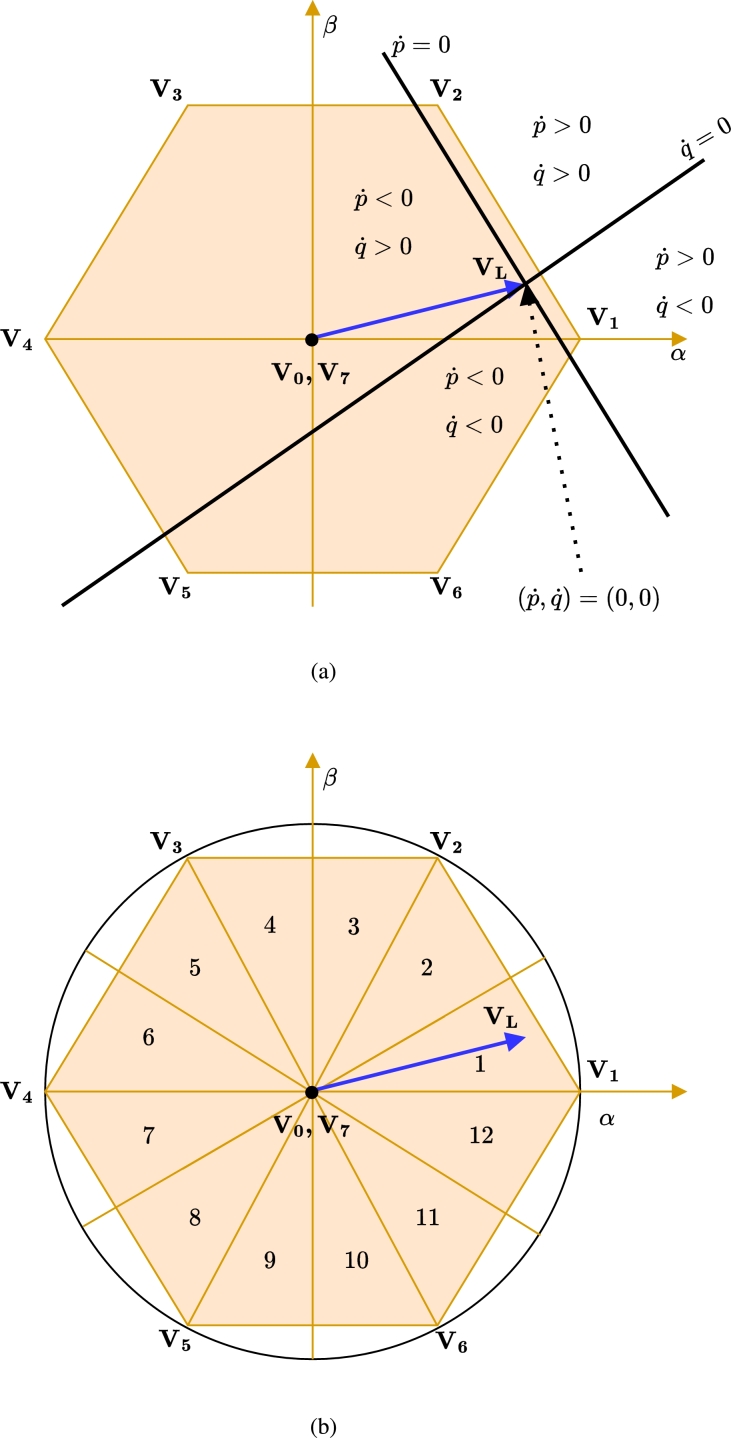
(a) Voltage Space Phasor structure in hexagonal plane and subspace (b) Division of hexagonal plane into 12- sector.

The shunt compensator switches $S_{1},S_{2}$, and $S_{3}$ have discrete values of 1 or 0. As a result, the voltage vector occupies a fixed position in the space as shown in Fig. 3a. The projection of the switching vector on the α−β plane is determined using below mathematical expression,

(2) $$v_{sh\alpha }=\sqrt{\frac{2}{3}}V_{dc}\left(S_{1}-\frac{S_{2}}{2}-\frac{S_{3}}{2}\right)v_{sh\beta }=\sqrt{\frac{1}{2}}V_{dc}\left(S_{2}-S_{3}\right)$$

Assuming load voltage vector components $v_{L\alpha }$ and $v_{L\beta }$ consist only of the fundamental positive sequence, we have:

(3) $$v_{L\alpha }=\sqrt{2}V_{ph}\cos(\omega_{e}t)v_{L\beta }=\sqrt{2}V_{ph}\sin(\omega_{e}t)$$

If all quantities are computed in the stationary reference frame, the instantaneous power equations are given by,

(4) $$\begin{array}{c} p_{sh}=\frac{3}{2}\left(v_{L\alpha }i_{f\alpha }+v_{L\beta }i_{f\beta }\right)\\ q_{sh}=\frac{3}{2}\left(v_{L\beta }i_{f\alpha }-v_{L\alpha }i_{f\beta }\right) \end{array}$$

Next, we differentiate Eq. (4) with respect to time,

(5) $$\begin{array}{c} \overset{˙}{p_{sh}}=\frac{3}{2}\left(v_{L\alpha }\overset{˙}{i_{f\alpha }}+i_{f\alpha }\overset{˙}{v_{L\alpha }}+v_{L\beta }\overset{˙}{i_{f\beta }}+i_{f\beta }\overset{˙}{v_{L\beta }}\right)\\ \overset{˙}{q_{sh}}=\frac{3}{2}\left(v_{L\beta }\overset{˙}{i_{f\alpha }}+i_{f\alpha }\overset{˙}{v_{L\beta }}-v_{L\alpha }\overset{˙}{i_{f\beta }}-i_{f\beta }\overset{˙}{v_{L\alpha }}\right) \end{array}$$

By substituting the expressions for $\overset{˙}{i_{f\alpha }}$ and $\overset{˙}{i_{f\beta }}$ from Eq. (1) and the load voltage derivatives from Eq. (3) and ignoring the effect of the resistance of the coupling inductor in Eq. (5), we have:

$$\begin{array}{c} \overset{˙}{i_{f\alpha }}=\frac{v_{sh\alpha }}{L_{f}}-\frac{v_{L\alpha }}{L_{f}}\\ \overset{˙}{i_{f\beta }}=\frac{v_{sh\beta }}{L_{f}}-\frac{v_{L\beta }}{L_{f}} \end{array}$$

and

$$\overset{˙}{v_{L\alpha }}=-\omega_{e}\sqrt{2}V_{ph}\sin(\omega_{e}t)=-\omega_{e}v_{L\beta }\;\overset{˙}{v_{L\beta }}=\omega_{e}\sqrt{2}V_{ph}\cos(\omega_{e}t)=\omega_{e}v_{L\alpha }$$

Substituting these values into Eq. (5) and rearranging terms, the power variation rates are obtained as

(6) $$\overset{˙}{p_{sh}}=\frac{3}{2L_{f}}\left[\left(v_{L\alpha }^{2}+v_{L\beta }^{2}\right)-\left(v_{sh\alpha }v_{L\alpha }+v_{sh\beta }v_{L\beta }\right)\right]-\omega_{e}q_{sh}$$

(7) $$\overset{˙}{q_{sh}}=\frac{3}{2L_{f}}\left[v_{sh\alpha }v_{L\beta }-v_{sh\beta }v_{L\alpha }\right]+\omega_{e}p_{sh}$$

These expressions are evaluated using values of Eq. (2) for determination of the power variation rates and thereby the switching vector for DPC in the shunt compensator.

#### 3.1.2 Selection of sliding surfaces

In the shunt-compensator, IARP contain both oscillating and average components. To effectively compensate for harmonics and reactive power, the controller must track both components. The primary objective of the SMC-DPC is to ensure that power errors slide along predefined surfaces of the IARP as defined in Eq. (8).

(8) $$S_{1}={\left[\begin{array}{cc} S_{p}\; & S_{q} \end{array}\right]}^{T}$$

Where

$$\begin{array}{c} S_{p}=k_{p}\left(p_{sh}^{⁎}-p_{sh}\right)\\ S_{q}=k_{q}\left(q_{sh}^{⁎}-q_{sh}\right) \end{array}$$

Since power error is chosen as the sliding surface, its first-order derivative, i.e. the rate of change of power error $\left(\overset{˙}{p},\overset{˙}{q}\right)$ must be considered to attain a solution that satisfies the control requirement. The existence of $\left(\overset{˙}{p},\overset{˙}{q}\right)$ in the subspace of hexagonal plane of converter voltage space phasor structure is shown in Fig. 3a. The rate of power variation depends on the position of the load voltage vector and the chosen voltage vector of the VSI. Based on the effect of power variation, the trajectory of load voltage space phasor is divided into 12 equidistant sectors as shown in Fig. 3b.

During the control sample time, the value of instantaneous active power reference $p_{sh}^{⁎}$ and the instantaneous reactive power reference $q_{sh}^{⁎}$ is held constant. Therefore, the projection of system motion in subspace is given by Eq. (6) and Eq. (7) where,

(9) $$\begin{array}{c} \overset{˙}{S_{p}}=-k_{p}\overset{˙}{p_{sh}}\\ \overset{˙}{S_{q}}=-k_{q}\overset{˙}{q_{sh}} \end{array}$$

In SMC-DPC, the Lyapunov direct method is used to derive conditions that ensures the system stability and ultimately drive the state (power errors) to the equilibrium point in the $\left(\overset{˙}{p},\overset{˙}{q}\right)$ subspace. The scalar function V(t) in the Lyapunov method is defined as,

(10) $$V(t)=\frac{1}{2}S_{1}^{T}S_{1}$$

Hence, the derivative of the Eq. (10) will be,

(11) $$\overset{˙}{V}(t)=S_{1}^{T}\overset{˙}{S_{1}}$$

where $\overset{˙}{S_{1}}$ is obtained using Eq. (9). The sliding surface will be asymptotically stable if the derivative of the Lyapunov function obtained using Eq. (11) is less than zero i.e. $\overset{˙}{V}(t)<0$. To ensure that the instantaneous power errors converge to zero, the inequality must be satisfied. Specifically, if $S_{p}>0$, the converter voltage vector should be selected such that $\overset{˙}{S_{p}}<0$ resulting in $S_{p}\overset{˙}{S_{p}}<0$. Similarly, if $S_{q}>0$, the converter voltage vector should be selected such that $\overset{˙}{S_{q}}<0$ resulting into $S_{q}\overset{˙}{S_{q}}<0$ and vice-versa. Since the voltage space phasor structure lies uniformly in the α−β plane, there will be at least one or more than one voltage vector shown in Fig. 3b, that fulfills this condition [51]. Therefore, the system stability is ensured provided the correct voltage vector of the VSI is applied for the control sample time.

#### 3.1.3 Determination of the LUT

Implementing the system can be further simplified if for a value of error $S_{p}>0$ and $S_{q}>0$, the voltage vector satisfies,

(12) $$sgn\left(S_{p/q}\right)=\left\{ \begin{array}{cc} 1 & S_{p/q}\geq h_{p/q}\\ \frac{S_{p/q}}{h_{p/q}} & \left|S_{p/q}\right|<h_{p/q}\\ 0 & S_{p/q}\leq h_{p/q} \end{array}\right.$$

where $h_{p/q}$ denotes the width of the boundary layer for instantaneous active/reactive respective power error. The appropriate width of the boundary layer eliminates the chattering problem in the system. When power error reaches the boundary layer on either side, the value is toggled from 1 to 0 or vice-versa. Based on the quantized error value of Eq. (12) and the position of the load voltage vector in a given sector, an appropriate voltage vector is selected from the LUT [52]. The choice of vector in Table 2 ensures minimum power variation and switching instants which in turn reduces losses in the compensator [52]. In this work, the value of power error, and therefore $S_{p}$ and $S_{q}$, is assumed to be zero to precisely track the reference powers.

**Table 2:** Switching Vector Selection Table /LUT of shunt compensator and series compensator for Voltage and Current Sector.

Sector $V_{L}\;or\;I_{s}$		Θ_1_	Θ_2_	Θ_3_	Θ_4_	Θ_5_	Θ_6_	Θ_7_	Θ_8_	Θ_9_	Θ_10_	Θ_11_	Θ_12_
*sgn*(*S*_*p*_)	*sgn*(*S*_*q*_)												
1	1	**V**_**1**_	**V**_**1**_	**V**_**2**_	**V**_**2**_	**V**_**3**_	**V**_**3**_	**V**_**4**_	**V**_**4**_	**V**_**5**_	**V**_**5**_	**V**_**6**_	**V**_**6**_
1	0	**V**_**2**_	**V**_**2**_	**V**_**3**_	**V**_**3**_	**V**_**4**_	**V**_**4**_	**V**_**5**_	**V**_**5**_	**V**_**6**_	**V**_**6**_	**V**_**1**_	**V**_**1**_
0	1	**V**_**7**_	**V**_**1**_	**V**_**0**_	**V**_**2**_	**V**_**7**_	**V**_**3**_	**V**_**0**_	**V**_**4**_	**V**_**7**_	**V**_**5**_	**V**_**0**_	**V**_**6**_
0	0	**V**_**7**_	**V**_**0**_	**V**_**0**_	**V**_**7**_	**V**_**7**_	**V**_**0**_	**V**_**0**_	**V**_**7**_	**V**_**7**_	**V**_**0**_	**V**_**0**_	**V**_**7**_

### 3.2 Control of series compensator

The control of the series compensator is further divided into the modeling of a series compensator connected to a matching transformer for the determination of power variation rates, selection of sliding surface, and determination of LUT from the observation of power variation rates.

#### 3.2.1 Modelling of series compensator

A series compensator of UPQC is a dual of the shunt compensator with added second-order dynamics. The analysis of series compensator shown in Fig. 2 reveals the following equations:

(13) $$v_{se\alpha }=L_{se}\overset{˙}{i_{se\alpha }}+v_{inj\alpha }\;v_{se\beta }=L_{se}\overset{˙}{i_{se\beta }}+v_{inj\beta }$$

(14) $$i_{s\alpha }=i_{se\alpha }-C_{se}\overset{˙}{v_{inj\alpha }}\;i_{s\beta }=i_{se\beta }-C_{se}\overset{˙}{v_{inj\beta }}$$

Here, $L_{se}$ is the coupling inductor of series compensator, $i_{se\alpha }$ and $i_{se\beta }$ are components of current through coupling inductor of series compensator and $v_{se\alpha }$ and $v_{se\beta }$ are components of voltage of the series compensator whereas the load source current components $i_{s\alpha }$ and $i_{s\beta }$ is assumed to have fundamental positive sequence and is in phase with the voltage i.e.

(15) $$i_{s\alpha }=\sqrt{2}I_{s}\cos(\omega_{e}t)i_{s\beta }=\sqrt{2}I_{s}\sin(\omega_{e}t)$$

The IARP injected by the series compensator are defined as,

(16) $$\begin{array}{c} p_{se}=\frac{3}{2}\left(v_{inj\alpha }i_{s\alpha }+v_{inj\beta }i_{s\beta }\right)\\ q_{se}=\frac{3}{2}\left(v_{inj\beta }i_{s\alpha }-v_{inj\alpha }i_{s\beta }\right) \end{array}$$

Differentiating the Eq. (16) with respect to time,

(17) $$\begin{array}{c} \overset{˙}{p_{se}}=\frac{3}{2}\left(v_{inj\alpha }\overset{˙}{i_{s\alpha }}+i_{s\alpha }\overset{˙}{v_{inj\alpha }}+v_{inj\beta }\overset{˙}{i_{s\beta }}+i_{s\beta }\overset{˙}{v_{inj\beta }}\right)\\ \overset{˙}{q_{se}}=\frac{3}{2}\left(v_{inj\beta }\overset{˙}{i_{s\alpha }}+i_{s\alpha }\overset{˙}{v_{inj\beta }}-v_{inj\alpha }\overset{˙}{i_{s\beta }}-i_{s\beta }\overset{˙}{v_{inj\alpha }}\right) \end{array}$$

Substituting Eq. (14) and Eq. (15) in the Eq. (17) and simplifying, we obtain following equation for instantaneous power rates in the series compensator

(18) $$\overset{˙}{p_{se}}=\frac{3}{2C_{se}}\left[\left(i_{s\alpha }^{2}+i_{s\beta }^{2}\right)-\left(i_{se\alpha }i_{s\alpha }+i_{se\beta }i_{s\beta }\right)\right]-\omega_{e}q_{se}$$

(19) $$\overset{˙}{q_{se}}=\frac{3}{2C_{se}}\left[i_{se\alpha }i_{s\beta }-i_{se\beta }i_{s\alpha }\right]+\omega_{e}p_{se}$$

In the case of the series compensator, the injected voltage will be zero except in the case of voltage sag or unbalance where the converter has to inject the voltage in phase or quadrature as determined by the algorithm. Subsequently, Eq. (13) is redundant under normal operating conditions. Hence, the instantaneous power requirements during the normal operating condition is zero.

#### 3.2.2 Selection of sliding surfaces

The sliding surface function for the series compensator with second order dynamics is defined by Eq. (20),

(20) $$S_{2}={\left[\begin{array}{cc} S_{u}\; & S_{v} \end{array}\right]}^{T}$$

where

$$\begin{array}{c} S_{u}=k_{u}\left(p_{se}^{⁎}-p_{se}\right)+\left(\overset{˙}{p_{se}^{⁎}}-\overset{˙}{p_{se}}\right)\\ S_{v}=k_{v}\left(q_{se}^{⁎}-q_{se}\right)+\left(\overset{˙}{q_{se}^{⁎}}-\overset{˙}{q_{se}}\right) \end{array}$$

When the system is operating in sliding mode, the sliding function attains zero value and hence,

(21) $$\begin{array}{c} \overset{˙}{p_{se}}=k_{u}\left(p_{se}^{⁎}-p_{se}\right)\\ \overset{˙}{q_{se}}=k_{v}\left(q_{se}^{⁎}-q_{se}\right) \end{array}$$

The time derivative of Eq. (21) is written as,

(22) $$\begin{array}{c} {\overset{˙}{S}}_{u}=-k_{u}^{2}\left(p_{se}^{⁎}-p_{se}\right)-\overset{¨}{p_{se}}\\ {\overset{˙}{S}}_{v}=-k_{v}^{2}\left(q_{se}^{⁎}-q_{se}\right)-\overset{¨}{q_{se}} \end{array}$$

The investigation of Eq. (22) after substituting the value of Eq. (18) and Eq. (19) reveals that the derivative of the sliding surface depends on the source current, converter voltage vector, and voltage to be injected in series with the line. In addition to this, the solution of Eq. (21) ensures that under dynamic conditions, the instantaneous power errors $e_{p}(t)=p_{se}^{⁎}-p_{se}$ and $e_{q}(t)=q_{se}^{⁎}-q_{se}$ in the series compensator follow,

(23) $$e_{p/q}(t)=e_{p/q}(0)\exp\left(-k_{p/q}t\right)$$

**Figure 4:**
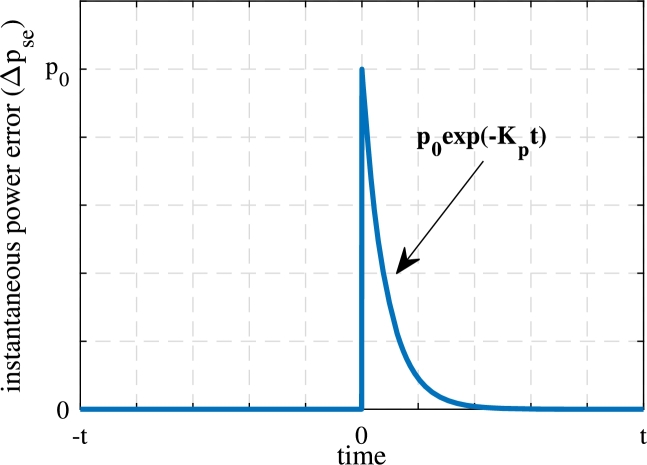
Change in instantaneous power error during the dynamic condition in the proposed method.

Where $1/k_{p/q}$ is the rate of convergence of active or reactive power at which the system converges to the zero error asymptotically. The solution obtained in Eq. (23) ensures that the instantaneous power delivered by the series compensator tracks the reference irrespective of the value of unbalance, the voltage sag and the parameters of the coupling inductor and capacitor. The instance of dynamic change is demonstrated in Fig. 4 during the onset of sag. The instantaneous active power error slides along the surface. As the sag is introduced, the instantaneous active power requirement of the series compensator changes. Due to this, the error attains a new value $p_{0}$ at the instant of sag considered as t=0 s. In the next sample time, the controller takes remedial action, thus the power error decreases asymptotically and again reduces to zero. This phenomena will be further verified in the results section.

#### 3.2.3 Determination of the LUT

The LUT for series compensator is designed based on the logic applied to the shunt compensator except that the source current space phasor is taken as the reference and is divided into 12 sectors. In this sense, the control of the series compensator is assumed as dual of the shunt compensator. It also leads to the fact that the control in SMC-DPC does not require information on the parameters during the processing of the algorithm. Hence, the variation in parameters does not affect the operation of the compensator.

## 4 Reference algorithm for UPQC

**Figure 5:**
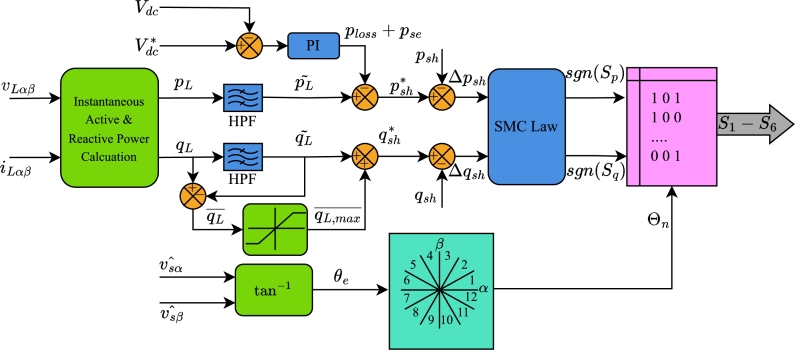
Control Algorithm for shunt compensator of UPQC.

**Figure 6:**
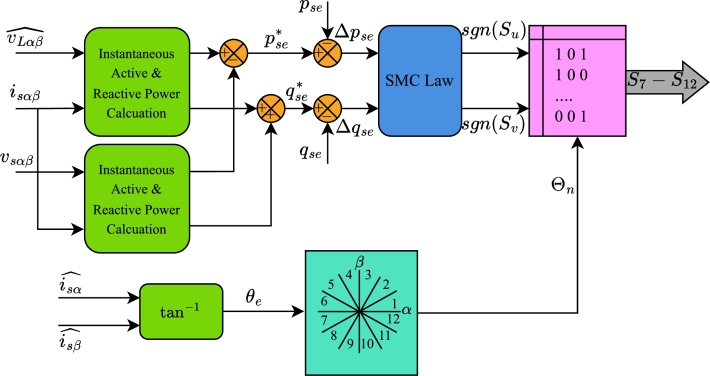
Proposed Control Algorithm for Series Compensator as a dual shunt compensator.

Fig. 5 illustrates the control block diagram for the shunt compensator, while Fig. 6 depicts the control block diagram for the series compensator of the UPQC. To maintain the simplicity of the method, the algorithm is developed entirely within stationary reference frame. The measured voltage and current values are first transformed into their α−β components using Clarke transformation. These transformed values are then fed into the proposed reference algorithm. The algorithm calculates the IARP for both compensators based on the components of the load voltage and current in the stationary reference frame. For example, the IARP of the load is derived from the load currents and the estimated voltages.

(24) $$\begin{array}{c} p_{L}=\overset{ˆ}{v_{L\alpha }}i_{L\alpha }+\overset{ˆ}{v_{L\beta }}i_{L\beta }\\ q_{L}=\overset{ˆ}{v_{L\beta }}i_{L\alpha }-\overset{ˆ}{v_{L\alpha }}i_{L\beta } \end{array}$$

A high-pass filter (HPF) is used to separate harmonic components from the fundamental components. Regarding instantaneous active power, the instantaneous active power generated by the DC link is included into the harmonic component. In addition to the power loss component $p_{loss}$, the DC link voltage controller also generates the active power $p_{se}$ required for series compensation. Conversely, the maximum rating of the shunt compensator influences the regulation of reactive power demand. Applying the SMC law to the error of IARPs determines the values of $sgn(S_{p})$ and $sgn(S_{q})$ for input to the LUT.

The estimated source voltages $\overset{ˆ}{v_{s\alpha }}$ and $\overset{ˆ}{v_{s\beta }}$ are necessary for calculating the grid angle, which in turn determines the sector $\Theta_{n}$. The algorithm determines the switching state of the shunt compensator based on the value of the sector and response of the SMC law.

In contrast, the algorithm for the series compensator is far less complex. The reference active and reactive power are determined by subtracting the measured load power from the estimated load power. These powers are thereafter compared to the actual instantaneous powers injected by the series compensator. The errors $\Delta p_{se}^{⁎}$ and $\Delta q_{se}^{⁎}$ between reference and actual IARP are fed to the SMC law block. Based on the output of the SMC law i.e. $sgn(S_{u})$ and $sgn(S_{v})$ and the sector of the source current, the LUT generates switching signals $S_{7}-S_{12}$ for the series compensator. These algorithms ensure that only average active power flows from the source to the load.

The following subsections address the crucial elements of the proposed algorithm, which include Control of DC link voltage, Positive Sequence Voltage Detector, and estimation of load voltage and source currents.

### 4.1 Control of DC link voltage

A DC link voltage controller is employed to control the voltage across the common DC link and to maintain the voltage across the DC link capacitor to ensure satisfactory operation of the system [53]. Here, the shunt compensator will effectively compensate for harmonics and reactive power if the following criteria of Eq. (25) are met

(25) $$V_{DC}\geq \frac{2}{m_{a}}\sqrt{\frac{2}{3}}V_{LL}$$

Where $V_{DC}$ is the magnitude of DC link voltage, $m_{a}$ is the modulation index of the converter, and $V_{LL}$ is the line voltage. The PI controller generates the $p_{dc}$ component in the algorithm. Assuming the value of controller as $k_{pd}$ and $k_{id}$ respectively, value of $p_{dc}$ is given by Eq. (26)

(26) $$p_{dc}(k)=p_{loss}(k)+p_{se}(k)p_{dc}(k)=p_{dc}\left(k-1\right)+k_{pd}\left(\Delta V_{dc}(k)-\Delta V_{dc}(k-1)\right)+k_{id}\Delta V_{dc}(k)$$

### 4.2 Positive sequence voltage detector

An accurate extraction of the positive sequence component from the grid voltage is crucial, especially considering the presence of unbalance conditions and harmonics in the source voltage. To ensure satisfactory operation in the stationary reference frame, a robust and adaptive positive sequence estimator is employed. Unlike conventional PLL methods, which can struggle under grid variations, this adaptive approach offers superior dynamic response and remains resilient to both grid voltage variations and frequency deviations. In this work, the transformation is applied to the source voltage with the frequency of $\omega_{0}$ such that

(27) $${\overset{ˆ}{v_{s}}}_{\alpha \beta }\left(\omega t\right)=\int e^{-\left(K_{\sigma }-j\omega_{0}\right)t}v_{s\alpha \beta }\left(\omega t\right)\cdot d\left(\omega t\right)$$

**Figure 7:**
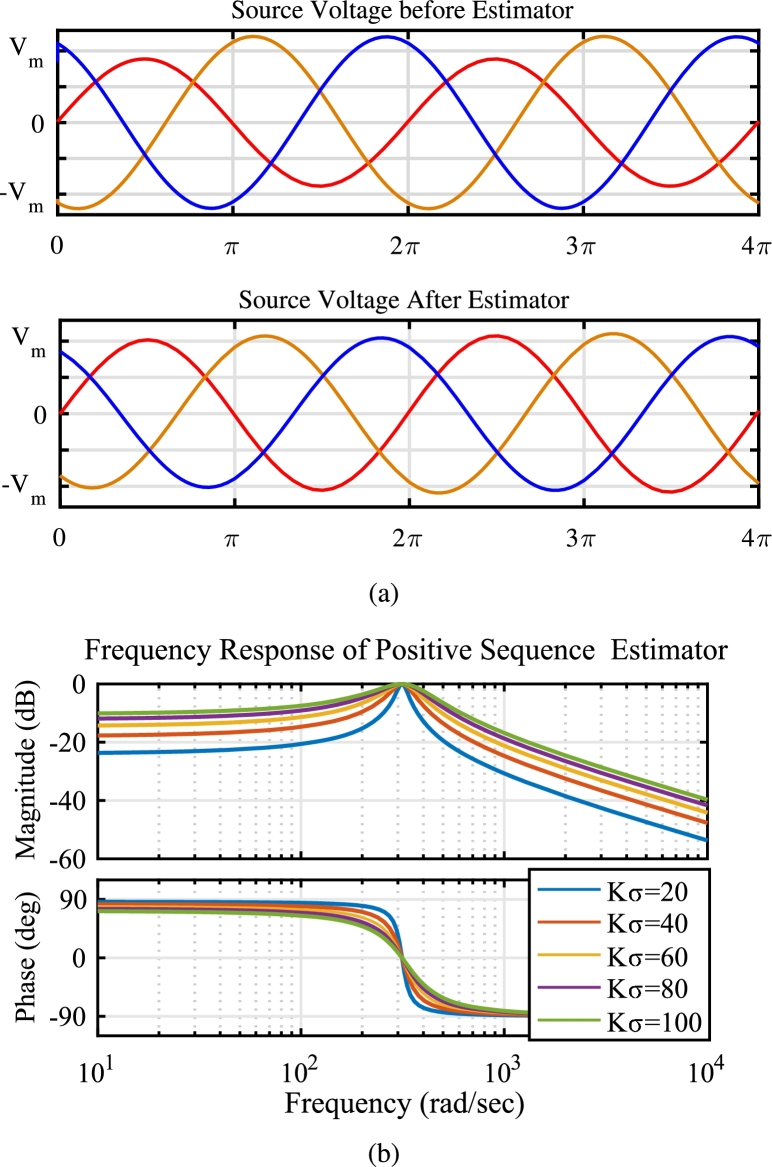
(a) Performance of Positive Sequence Voltage Detector during unbalance (b) Frequency response of Positive Sequence Voltage Estimator at different values of constant *K*_*σ*_.

Fig. 7a illustrates the performance of the implemented positive sequence extraction technique under unbalanced grid voltage conditions. The transformation parameters of Eq. (27), namely the tuned frequency ($\omega_{0}$) and sharpness constant ($K_{\sigma }$), were determined based on the frequency response analysis depicted in Fig. 7b. These parameters were carefully chosen to achieve a unity gain at the fundamental frequency, ensuring accurate extraction of the positive sequence component. It is important to note that the sharpness constant ($K_{\sigma }$) plays a crucial role in this transformation. While a higher $K_{\sigma }$ value enhances the selectivity of the desired frequency component, it can also impact the transient response of the system. The smaller value of i.e. $K_{\sigma }=20$ suggests faster dynamic response at the cost of stability whereas the higher value of $K_{\sigma }=100$ provides better stability but slower dynamic response. In this work, $K_{\sigma }=60$ is chosen that trades-off between stability and dynamic response of the system whereas the value of $\omega_{0}$ is chosen as 314.16 rad/sec.

The simplification of expression Eq. (27) and conversion into the discrete domain with backward Euler's method yields the expression for obtaining estimated fundamental voltage,

(28) $$\begin{array}{c} \overset{ˆ}{v_{s\alpha }}\left(k\right)=\overset{ˆ}{v_{s\alpha }}\left(k-1\right)+K_{\sigma }\left[v_{s\alpha }\left(k\right)-v_{s\alpha }\left(k-1\right)\right]-\omega_{0}\overset{ˆ}{v_{s\beta }}\left(k\right)\\ \overset{ˆ}{v_{s\beta }}\left(k\right)=\overset{ˆ}{v_{s\beta }}\left(k-1\right)+K_{\sigma }\left[v_{s\beta }\left(k\right)-v_{s\beta }\left(k-1\right)\right]-\omega_{0}\overset{ˆ}{v_{s\alpha }}\left(k\right) \end{array}$$

Based on these estimated voltages from Eq. (28), the angular information of the voltage is obtained as

$$\theta_{e}=\arctan\left(\frac{{\overset{ˆ}{v}}_{s\beta }(k)}{{\overset{ˆ}{v}}_{s\alpha }(k)}\right)$$

### 4.3 Estimation of load voltage and source current

Although the information obtained from Eq. (28) is adequate for the operation of the series compensator under unbalanced and distorted conditions, the operation under sag requires the calculation of the reference voltage to be injected. The reference injected voltage is calculated from

(29) $$\begin{array}{c} v_{inj,\alpha }^{⁎}=\sqrt{2}V_{ph}^{⁎}\cos\theta_{e}-\overset{ˆ}{v_{s\alpha }}\\ v_{inj,\beta }^{⁎}=\sqrt{2}V_{ph}^{⁎}\sin\theta_{e}-\overset{ˆ}{v_{s\beta }} \end{array}$$

The series injected voltage obtained from above Eq. (29) is in phase with the source current. From the above-obtained quantities, the load voltage and source current are estimated using Eq. (30),

(30) $$\begin{array}{c} \overset{ˆ}{v_{L\alpha }}=\overset{ˆ}{v_{s\alpha }}+v_{inj\alpha }^{⁎}\\ \overset{ˆ}{v_{L\beta }}=\overset{ˆ}{v_{s\beta }}+v_{inj\beta }^{⁎}\\ \overset{ˆ}{i_{s\alpha }}=i_{L\alpha }+i_{f\alpha }\\ \overset{ˆ}{i_{s\beta }}=i_{L\beta }+i_{f\beta } \end{array}$$

These estimated load voltages and source currents are used for the calculation of active and reactive power. For the series compensator, the instantaneous reference values of active and reactive power are defined by calculations similar to that of Eq. (24) where the estimated voltage is replaced by the estimated current and the load current is replaced by the injected voltage.

The value of the reference power thus obtained is compared with the instantaneous values of powers, which are calculated from measured values by sensors, and the error is applied to the switching law of the SMC which selects the voltage vector of the series compensator. For the determination of the voltage vector, Eq. (12) equally applies.

## 5 Determination of parameters of UPQC

The overall rating of UPQC depends on the independent ratings of the shunt compensator and Series compensator. The independent ratings are calculated as follows [54], [45]:

Given a rated system voltage of 230 V, a supply frequency of 50 Hz, and a load with a total rating of 17.9 kVA, the system introduces a distortion of 15.93%. At t=0.1s, the power factor of system is 0.66, necessitating the shunt compensator to compensate for both reactive power and harmonics. Simultaneously, a voltage sag in the source voltage increases the demand for power from the shunt compensator, as the DC link cannot handle the active power requirement. Consequently, the overall UPQC rating is determined by the total power needed for reactive power compensation and the active power required during voltage sag.

The rating of the series compensator during a voltage sag of 0.3 pu is calculated as:

$$S_{se}=\sqrt{3}\times 400\times 0.3\times 17.23=3.57\;\text{kVA}$$

The rating of the shunt compensator is calculated based on three factors: reactive power compensation, harmonic compensation, and power requirement during voltage sag i.e.

$$S_{sh}=\sqrt{3}\times V_{LL}\times \sqrt{I_{p,sag}^{2}+I_{h}^{2}+I_{q}^{2}}$$

Assuming a line voltage rating of $V_{LL}=400\;\text{V}$, the kVA rating of SAPF is 14 kVA, calculated as follows:

$$S_{sh}=\frac{3\times 230\times \sqrt{{\left(5.17\right)}^{2}+{\left(4.08\right)}^{2}+{\left(19.18\right)}^{2}}}{1000}$$

This shows that the overall shunt compensator rating increases due to the active power requirement during voltage sag. Furthermore, the overall rating is calculated considering overloading factor. Thus, the rating of UPQC is considered as 20 kVA.

Considering maximum instantaneous active power variation occurring on the application zero vector from the Eq. (6), the minimum inductor value is calculated as [44],

$$L_{f}=\frac{3V_{ph}^{2}\times T_{s}}{2\Delta p_{sh}}$$

where the sample time $T_{s}=10\;\mu \text{s}$ and $\Delta p_{sh}=320\;\text{W}$. This value is critical to maintaining the instantaneous active and/or reactive power errors within band limits. As the selection of this value will result in the frequent crossing of boundaries for the nonlinear controller, a value of $L_{f}=5\;\text{mH}$ is selected. Similarly, the value of $L_{se}=4\;\text{mH}$ is selected for the series compensator.

The DC link capacitor value is based on the dynamic change in the system, allowing for a 25% change in the system voltage. The capacitor value is calculated as,

$$C_{dc}=\frac{4\pi \times n\times S_{sh}}{\omega_{e}\left({\left(1.125V_{dc}\right)}^{2}-{\left(0.875V_{dc}\right)}^{2}\right)}=3630\;\mu \text{F}$$

Where *n* is the number of fundamental cycles of supply within which the DC link must be restored and the value is taken as 3. It is not possible to match the calculated value in the physical configuration. Hence, four capacitors of 4700μF in a series-parallel combination with two capacitors each are assumed in the proposed system.

The value of the ripple filter is typically chosen so that it is tuned to half the switching frequency of the converter to reduce the switching ripples at the PCC. Assuming the switching frequency of 8kHz and effective reactance $X_{cf}$ of 1.5Ω at half the switching frequency,

$$C_{f}=\frac{1}{\pi f_{sh}X_{cf}}=26.52\;\mu \text{F}$$

The value of the ripple filter is taken as 25μF which is the nearest value of the power electronic film capacitor. In addition to this, the calculated value offers a sufficiently high impedance at the fundamental frequency. For the proposed system, it is assumed that two capacitors of 50μF are connected in series to meet the voltage rating. In addition to this, the damping resistors of 1.1Ω are connected in series with the capacitors to avoid resonance phenomena.

## 6 Simulation results and discussions

To validate the effectiveness of the proposed method, a simulation is conducted in the MATLAB Simulink® environment using Sim Power System${}^{TM}$ Toolbox. The system parameters are detailed in Table 3. A comparative performance evaluation is carried out, comparing the proposed method again two widely used and well established control methods, SMC and Linear Controller with PI. The simulation initializes with pre-charged DC link and activation of shunt and series compensators. The dynamics of the system are defined as follows:

• A nonlinear load having THD of 25.8% is connected to the load side.
• At t=0.1 s, an unbalanced voltage with an imbalance of 20% is introduced in the system. The unbalance must be compensated by the series compensator and hence the remedial action of series compensator is effectively introduced at the instant t=0.1 s.
• At t=0.15 s, the unbalance is removed from the system, returning the system to balanced condition.
• At t=0.2 s, a symmetrical sag of 30% magnitude occurs in all phases and lasts 5 cycles. This event necessitates dynamic compensation by the series compensator.
• At t=0.4 s, a step change in load is introduced where the demand of non-linear current is increased by 80% thus resulting in more THD with 15.93%.

**Table 3:** Parameters of Simulation.

Sr No.	Parameter/ Description	Value
1	Source Voltage	230 V
2	**Linear Load:**	
	Load Resistance	10 Ω
	Load Inductance	35 mH
	**Non-linear load:**	
	(Uncontrolled Rectifier)	
	DC Load Resistance	50 Ω
	DC Load Inductance	20 mH
3	**Shunt Compensator Ripple Filter:**	
	Coupling Inductor *L*_*f*_	5 mH
	Filter Capacitor *C*_*f*_	25 μF
4	**Series Compensator Ripple Filter:**	
	Coupling Inductor *L*_*se*_	4 mH
	Filter Capacitor *C*_*se*_	25 μF
5	**Series Transformer:**	
	Turns Ratio	1:1
	Leakage Inductance on each side	1 mH
	Winding Resistance on each side	0.2 Ω
6	DC link Capacitance	4700 μF
7	DC link Voltage	680 V

The subsequent sections detail analysis of simulation results under different operating conditions.

### 6.1 Case-1: compensation of harmonics and reactive power

**Figure 8:**
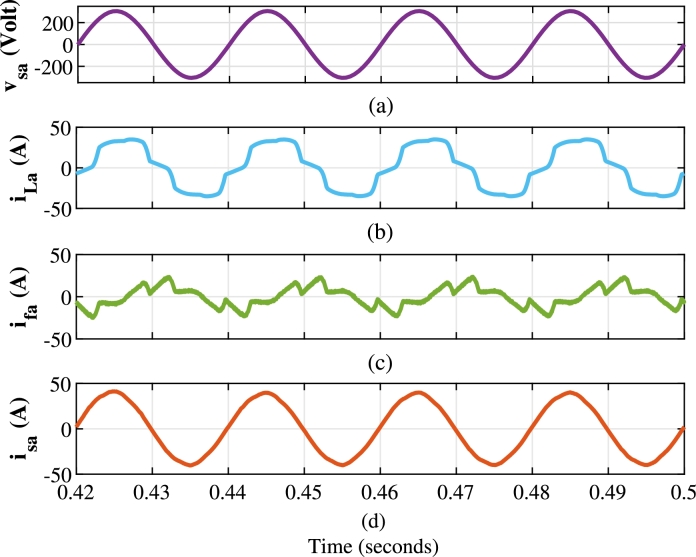
Performance of shunt compensator with the proposed method for one phase (a) Source Voltage *v*_*sa*_ (volt) (b) Load Current *i*_*La*_ (Amp) (c) Compensating Current *i*_*fa*_ (Amp) (d) Source Current *i*_*sa*_ (Amp).

**Figure 9:**
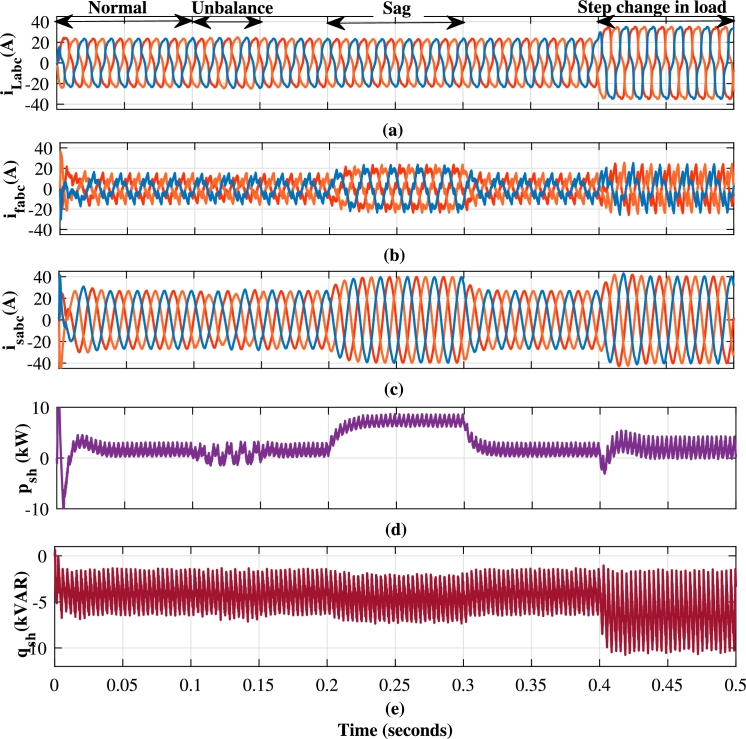
Performance of Shunt Compensator controlled with Sliding Mode DPC (a) Load Current *i*_*Labc*_ (A) (b) Compensating Current *i*_*fabc*_ (A) (c) Source Current *i*_*sabc*_ (A) (d) Instantaneous Active Power supplied by Shunt Compensator *p*_*sh*_ (kW) (e) Instantaneous reactive power supplied by Shunt Compensator *q*_*sh*_ (kVAR).

**Figure 10:**
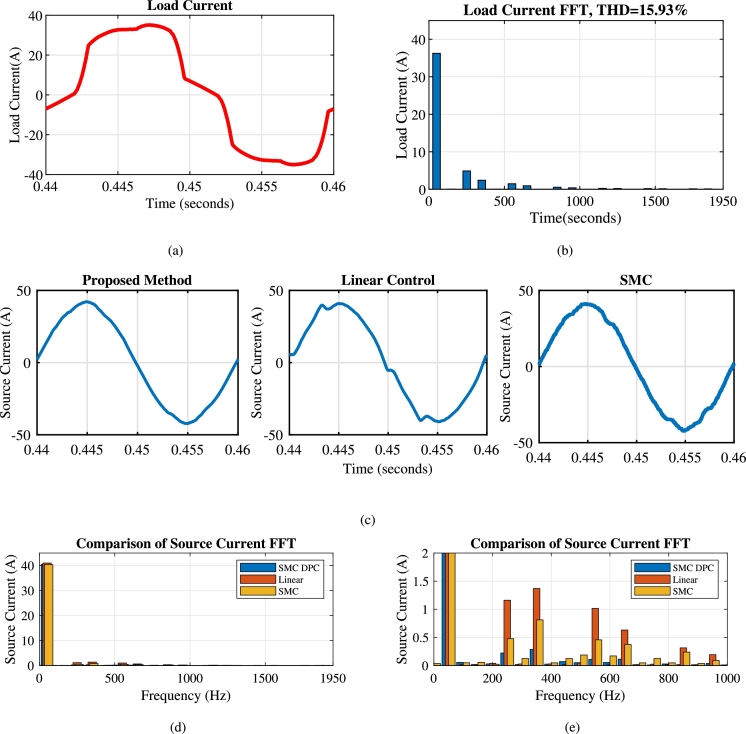
(a) Load Current Waveform (b) FFT spectrum of load current (c) Source current THD after compensation of shunt compensator with a different method (d) FFT of different methods (e) Zoomed view of FFT spectrum.

This section analyzes capability of the shunt compensator in mitigation of harmonics and reactive power. As depicted in Fig. 8(b), Fig. 10(a) and Fig. 10(b), the load current has a THD of 15.93% during t=0.4 s to t=0.5 s. The shunt compensator effectively mitigates harmonics through compensating current of Fig. 8(c). This results into the sinusoidal current as evidenced in Fig. 8(d), which is in phase with the source voltage of Fig. 8(a). Fig. 9 further illustrates the performance of shunt compensator during various scenarios. Fig. 9(a), Fig. 9(b) and 9(c) show the three phase loads current, compensating current and source current respectively. The oscillating component of IARP injected by shunt compensator shown in Fig. 9(d) and Fig. 9(e) respectively, eliminate harmonic currents. At the same time, the average component compensates for reactive power demand, achieving a near-unity power factor.

While the series compensator injects active power into the system during sag, the same value of active power must be drawn from the shunt compensator to maintain the voltage across the DC link. This has resulted in an increase of the source currents as shown in Fig. 9(c). The dynamic performance of the compensator is limited by the high-pass filter and performance of the DC link controller, unlike the grid-connected converter. The FFT spectrums of the load current in Fig. 10(b) and the source current in Fig. 10(d) and Fig. 10(e) validate the effectiveness of the shunt compensator. The source currents of proposed method, linear control method and SMC are reported in Fig. 10(c). As shown in Fig. 10(d), the proposed method achieves reduction in the source current THD to 1.52% at the moderate average switching frequency of 8 kHz in comparison to 2.31% and 5.11% of SMC and linear controller respectively.

Table 4 summarizes the performance comparison across various operating scenarios, further confirming the superior performance of the proposed method. The tabulated results highlight the consistent and significant performance advantage of the proposed method over existing state-of-the-art techniques, particularly in the context of shunt compensator operation.

**Table 4:** Comparison Table of Performance of the different controllers of UPQC under different operating conditions.

Time Duration	Source Current THD (%)			Load Voltage THD (%)		
	Proposed	SMC [37]	Linear Current	Proposed	SMC [37]	Linear Voltage
	Method		Controller [49]	Method		Controller [49]
Starting (0-0.1 s)	1.2	1.66	3.88	2.55	1.85	3.12
Unbalance (0.1-0.15 s)	2.8	3.70	4.91	2.5	2.06	3.4
Sag (0.2-0.3 s)	0.93	1.19	2.86	2.51	1.99	3.16
Post sag (0.3-0.4 s)	1.11	1.96	3.78	2.5	1.93	3.1
Step change (0.4-0.5 s)	1.53	2.33	5.17	2.6	2.37	4.18

### 6.2 Case-2: compensation of unbalance in source voltage

As highlighted in [55], non-linear loads, including those found in Uninterruptible Power Supplies, can exhibit “single-phasing” behavior under significant source voltage unbalance. This phenomenon leads to uneven loading across phases, with one phase potentially experiencing zero loading while the remaining phases endure increased current distortion. Maintaining load voltage within acceptable standards becomes paramount in such situations.

**Figure 11:**
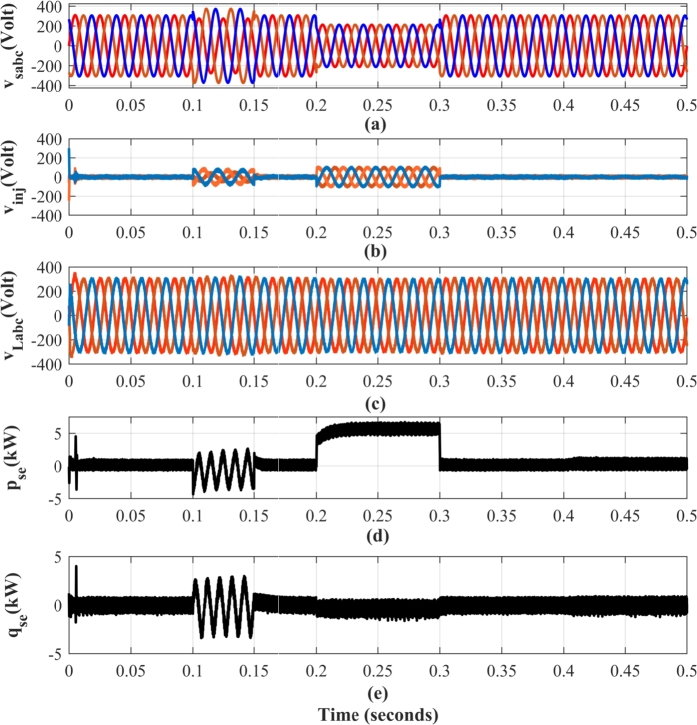
Performance of UPQC with proposed method for simultaneous compensation of unbalanced and voltage sag (a) Source Voltage *v*_*s*,*abc*_ (Volt)(b) Injected Voltage *v*_*inj*,*abc*_ (Volt) (c) Load Voltage *v*_*L*,*abc*_ (Volt) (d) Instantaneous Active Power supplied by series compensator *p*_*se*_ (kW) (e) Instantaneous reactive power supplied by series compensator *q*_*se*_ (kVAR).

To replicate this real-world scenario, a 20% voltage imbalance is introduced into the source voltage from t=0.1 s to t=0.15 s, as illustrated in Fig. 11(a). The series compensator effectively mitigates this unbalance by injecting IARP, thereby regulating the voltage in the system (Fig. 11(d) and Fig. 11(e)).

It is important to note that the series compensator injects only negative sequence currents to counteract the unbalance as shown in Fig. 11(b). As a result, the instantaneous power waveform associated with this compensation exhibits a frequency twice that of the fundamental frequency. While the reactive power compensation provided by the series compensator operates independently of the shunt compensator, the active power required for this compensation is drawn from the DC link. This is reflected in the operation of shunt compensator, leading to a slight distortion in the source current, as evident from the results presented in Table 4.

### 6.3 Case-3: compensation of sag

**Figure 12:**
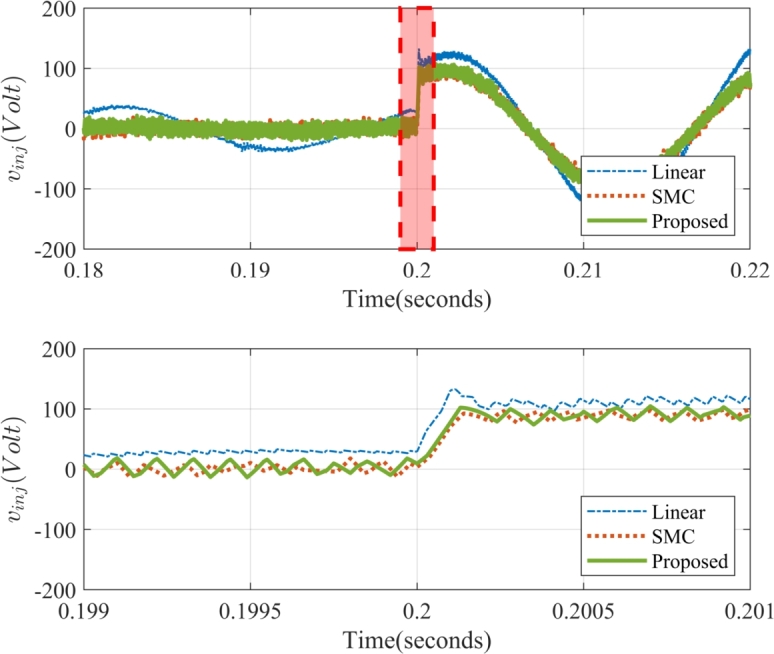
Dynamic Performance Comparison of Injected Voltage *v*_*inj*,*b*_ in the Series Compensator during Sag Compensation.

Fig. 11(a)-(e) illustrates the response of system to a 30% voltage sag introduced between t=0.2 s and t=0.3 s, clearly evident in the source voltage profile of Fig. 11(a). During the period, the series compensator effectively mitigates the sag by injecting the voltage in phase with the current. With this approach, the compensator injects only active power into the system. A minimal amount of reactive power is drawn by the coupled filters of series compensator. Fig. 12 provides a detailed view of the series compensator voltage injection profile at the onset of the voltage sag. Notably, the series compensator operates at a switching frequency of $f_{se}=5\;\text{kHz}$, sufficient for achieving both satisfactory compensation performance and a rapid dynamic response.

As illustrated in Fig. 12, the linear controller exhibits significant steady-state voltage errors and sluggish transient response. On the other hand, the proposed method demonstrates superior performance, achieving zero steady-state voltage errors and a faster settling time during dynamic voltage disturbances. This improvement in dynamic performance is achieved without the drawbacks associated with non-linear control techniques like SMC, such as chattering, ensuring a more stable and efficient operation of the UPQC.

**Figure 13:**
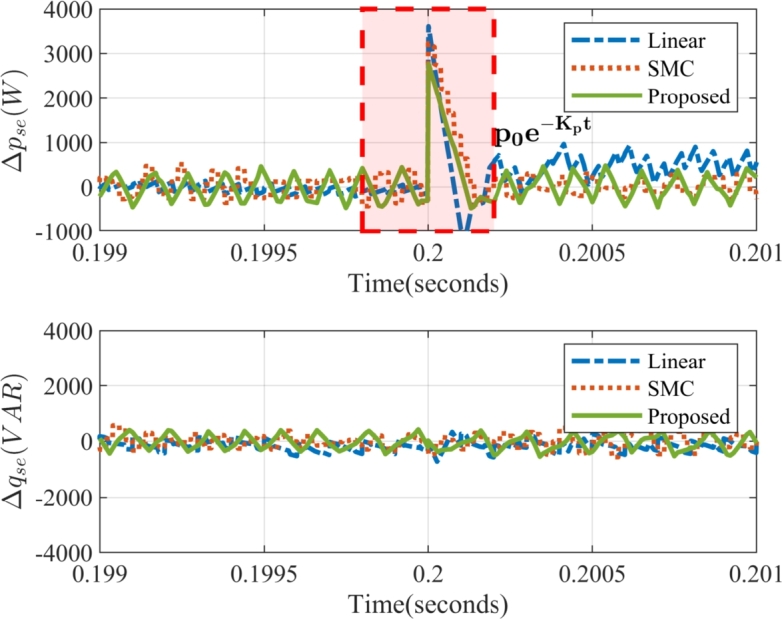
Instantaneous Active and Reactive Power Error in Series Compensator: Δ*p*_*se*_ (in W) – instantaneous active power error in series compensator, Δ*q*_*se*_ (in VAR)– instantaneous reactive power error in series compensator.

Fig. 13 presents a comparative analysis of the IARP tracking performance for the series compensators of the UPQC. The proposed control method demonstrates superior performance by effectively maintaining IARP errors within the predefined limits of 4% of the total converter power rating. While a slight violation of the active power error limit is observed at the initiation of the voltage sag, the controller implements remedial actions, effectively driving the error asymptotically towards zero. In contrast, both the conventional SMC and linear control methods exhibit prolonged settling times. This performance disparity stems from their inherent limitation in effectively decoupling IARP control. The ability of the proposed method to achieve precise and rapid IARP control, even during such conditions, underscores its superior performance and suitability for PQ enhancement in the grid.

**Figure 14:**
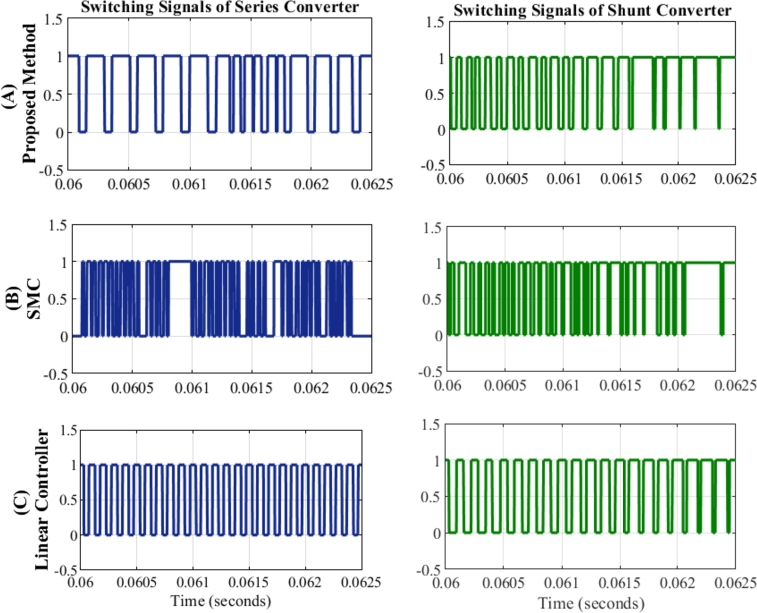
Comparison of Switching signals of series compensator and shunt compensator with (a) Proposed method (b) SMC (c) Linear Controller.

Fig. 14 provides a representation of switching performance of both converters. It is important to acknowledge that real-world voltage sags and swells are typically short duration and rare events. Hence, the UPQC spends a significant operational time injecting insignificant voltage into the system.

To facilitate a comparative assessment, the performance of various control methods is captured at t=0.06 s. The SMC exhibits large switching frequency variations and chattering, as evident in Fig. 14(b). Conversely, the linear controller as shown in Fig. 14(c) maintains fixed switching frequency, a desirable requirement for the semiconductor switches. At the same time, the variation in the switching frequency of the proposed method is confined within a narrow range as shown in Fig. 14(a). This is the most relevant finding of the proposed work and perhaps also the most significant since the nonlinear controller is often regarded as the cause of several problems in the grid.

Table 4 presents the THD observed in the load voltage waveform after compensation. A direct comparison with the SMC reveals a slightly higher THD level in the proposed method. This difference in performance can be attributed to the contrasting control priorities. SMC prioritizes precise tracking of individual injected voltages, irrespective of switching variations or their impact on other phases. This approach, while effective in minimizing THD, often comes at the expense of demerits mentioned earlier. In contrast, the proposed method adopts a more holistic approach, considering the switching states of the entire VSI unit.

Therefore, while the proposed controller may not achieve the same level of THD reduction as SMC, it successfully combines the desirable tracking capabilities of SMC with a significant reduction in its primary drawback. The advantages discussed above, particularly the reduction in switching-related issues, are key factors that make SMC-DPC a compelling alternative to conventional control methods.

The proposed UPQC controller effectively tracks IARP references with minimal delay – primarily limited to the sample time of controller. This rapid response is evident in the absence of overshoot or sluggish behavior, even during step changes in reference values. Furthermore, Fig. 11(c) demonstrates the ability of controller to maintain desirable load voltage profile. The load voltage remains free from common PQ issues such as notches, oscillations, or phase jumps, even during transient events like voltage sags.

### 6.4 DC link voltage profile of UPQC

**Figure 15:**
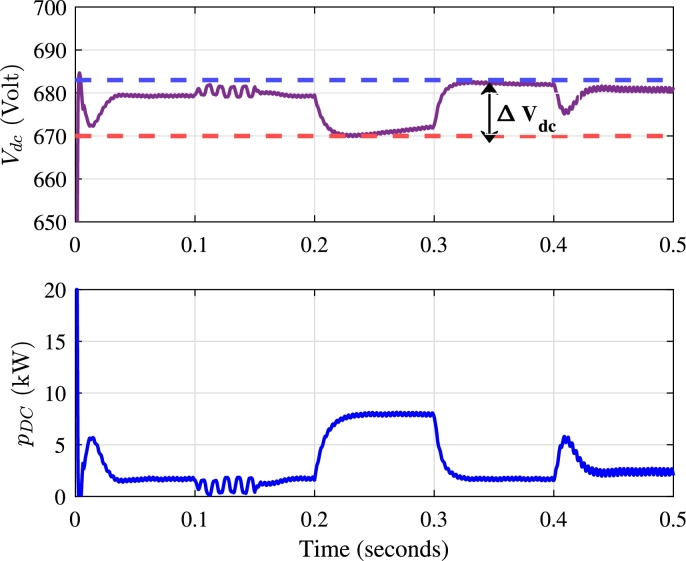
Performance of DC link voltage controller (a) DC link Voltage *V*_*dc*_ (Volts) (b) Active Power demanded from shunt compensator of UPQC *p*_*dc*_ (kW).

The performance of UPQC is significantly influenced by the voltage across the DC link. The DC link voltage magnitude governs the distribution of active and reactive power between the compensators. As illustrated in Fig. 15(a), the DC link voltage is effectively controlled during various compensation scenarios.

In steady-state conditions, where the average power requirement is zero, the DC link voltage magnitude remains stable. This stability is observed during the compensation of harmonics and unbalanced voltages. However, during dynamic events, such as current changes and voltage sags, the DC link controller responds by drawing active power from the shunt compensator to support the series compensator as shown in Fig. 15(b). The DC link voltage controller thus plays a crucial role in determining the amount of active power required during voltage sags.

The overall variation in the DC link voltage during and after a voltage sag is 1.91% as shown in Fig. 15(a), indicating the robustness of the DC link voltage controller in handling various power quality issues. During voltage sag, the shunt compensator consistently draws the same amount of active power. The demand for active power from the distribution line persists until the source voltage returns to its rated amplitude.

## 7 Performance of proposed method under weak and strong grid conditions

In the literature reported so far, the source impedance is considered negligible and conditions are ideal and suitable for the interconnection of the UPQC. The performance of a UPQC is significantly influenced by the characteristics of grid impedance. The present section aims to investigate the performance of UPQC under strong and weak grids. Table 5 outlines the resistance and inductance values used to model these grid types.

**Table 5:** Analysis of Percentage Real Power Line Loss with Integration of UPQC under Different Grid Conditions.

Strong PCC ($r_{s}=0.024\;\Omega$, $L_{s}=0.33\;\text{mH}$)					

	Proposed	SMC (%)	%increase	Linear	% increase
Cases	Method (%)	[37]	in losses	Controller [49]	in losses

Harmonic Compensation	0.9277	0.9287	0.11%	0.9327	0.54%
Voltage Sag	1.7908	1.7959	0.28%	1.8368	2.57%
Reactive Power Compensation	1.3273	1.3275	0.01%	1.3345	0.54%

Weak PCC ($r_{s}=0.24\;\Omega$, $L_{s}=3.3\;\text{mH}$)					

	Proposed	SMC (%)	%increase	Linear	% increase
Cases	Method (%)	[37]	in losses	Controller [49]	in losses

Harmonic Compensation	1.7938	1.8257	1.78%	1.8239	1.68%
Voltage Sag	3.4104	3.8781	13.71%	3.9063	14.54%
Reactive Power Compensation	1.5892	1.5899	0.04%	1.6483	3.72%

The analysis emphasizes the losses incurred within the distribution system. These losses encompass:

**Line Losses (**$P_{gl}$**):** Caused by the flow of source current through the line resistance ($r_{L}$). Eq. (31) quantifies these losses, taking into account the difference between load current ($i_{Lj}$) and compensator current ($i_{fj}$).

(31) $$P_{gl}=r_{L}\sum_{j=a,b,c}{\left(i_{Lj}-i_{fj}\right)}^{2}+P_{sw}$$

**Switching Losses (**$P_{sw}$**):** The switching losses of the converter, influenced by the efficiency of converter ($\eta_{conv}$), inverter voltage ($V_{i}$), and compensator current ($i_{fj}$). Eq. (32) defines these losses. For the sake of simplicity in analysis, the switching losses of the converter are assumed as 5% of the power rating at the time of operation.

(32) $$P_{sw}=(1-\eta_{conv})\sum_{j=a,b,c}V_{i}I_{fj}$$

**Harmonic Losses:** Additional losses stemming from harmonic currents present in the source current.

The primary objective while evaluating UPQC performance is minimizing line losses, as defined in Eq. (31). This analysis focuses on comparing line losses under three distinct compensation scenarios: (a) Harmonic Compensation (b) Reactive Power Compensation (c) Voltage Sag Compensation

As expected, a strong grid connection (SCR≈15), characterized by low source impedance, results in minimal impact on the UPQC performance. The compensator parameters dominate the system behavior, rendering the grid impedance relatively insignificant. However, when connected to a weak grid (SCR≈1.58) with higher source impedance, the controller adaptability becomes crucial. The proposed method demonstrates superior performance in this scenario, effectively reducing line losses compared to both the SMC and Linear Controller methods. During harmonic compensation with a weak grid, the proposed method achieves a line loss reduction of 1.78% compared to SMC and 1.68% compared to LCC. Furthermore, during voltage sag compensation, line losses are reduced by 13.71% compared to SMC and 14.54% compared to Linear Controller. Thus, the proposed UPQC control method demonstrates superior adaptability and performance across various grid conditions and compensation scenarios.

Although the work has numerous advantages in performance of the UPQC, there are notable limitations of the present work.

i Moderate variation in the switching frequency, which necessitates the use of larger inductors and/or capacitors in the design
ii Complexity in designing higher-order filters, such as LCL filters
iii The requirement for a large sampling frequency, which may limit the use of more complex control algorithms.

## 8 Conclusions

This research proposed an SMC-DPC for Unified Power Quality Conditioners to address the growing need for enhanced performance and adaptability in modern distribution systems. The proposed method demonstrated superior steady state and dynamic response, achieved decoupled control of active and reactive power and eliminated chattering problems in switching of converters. A novel load voltage estimation-based algorithm in stationary reference frame is developed for the SMC-DPC. The SMC-DPC with novel load voltage estimation algorithm has shown promising results for source current and load voltages under both strong and weak grid conditions. Furthermore, the method has demonstrated robust performance resulting in significant line loss reduction compared to conventional approaches, particularly in weak grid scenarios. These findings highlight its potential for enhancing power quality and efficiency in distribution systems. The future research will explore application of proposed algorithm for medium voltage distribution systems, optimization of the algorithm and its extension to wide range of PQ issues such as voltage harmonics and interruptions and compensation of PQ issues, with integration of RES.

## Funding statement

No funding was received for this research work.

## CRediT authorship contribution statement

**Tapankumar Trivedi:** Writing – review & editing, Writing – original draft, Methodology, Investigation, Formal analysis, Conceptualization. **Rajendrasinh Jadeja:** Writing – review & editing, Writing – original draft, Supervision, Resources, Methodology, Investigation, Conceptualization. **Praghnesh Bhatt:** Writing – review & editing, Writing – original draft, Supervision, Methodology, Investigation, Formal analysis, Conceptualization. **Chao Long:** Writing – review & editing, Writing – original draft, Formal analysis. **P. Sanjeevikumar:** Writing – review & editing, Writing – original draft, Formal analysis. **Amit Ved:** Writing – original draft, Investigation.

## Declaration of Competing Interest

The authors declare the following financial interests/personal relationships which may be considered as potential competing interests: Reports a relationship with that includes: Has patent pending to. If there are other authors, they declare that they have no known competing financial interests or personal relationships that could have appeared to influence the work reported in this paper.

## Data Availability

No data was used for the research described in the article.

## Appendix A

DC link Voltage Controller: $K_{pd}=150$, $K_{id}=2200$, Controller gain of Linear Current Controller: $K_{pc}=31.5$, $K_{ic}=1575$, Controller gain of Linear Voltage Controller: $K_{pv}=8$, $K_{iv}=120$, Sliding Mode Coefficients: $k_{p}=8000$, $k_{q}=8000$, $K_{u}=5000$, $K_{v}=5000$, Width of the boundary layer in SMC and SMC-DPC – 4%, Average Switching Frequency in proposed method: Shunt Compensator $f_{sh}=8\;\text{kHz}$, series compensator - $f_{se}=5\;\text{kHz}$.
